# Dysfunction and Morphological Involvement of Inner Macular Layers in Glaucoma

**DOI:** 10.3390/jcm13226882

**Published:** 2024-11-15

**Authors:** Vincenzo Parisi, Lucia Ziccardi, Sara Giammaria, Lucilla Barbano, Lucia Tanga, Manuele Michelessi, Gloria Roberti, Carmela Carnevale, Carmen Dell’Aquila, Mattia D’Andrea, Gianluca Manni, Francesco Oddone

**Affiliations:** 1 IRCCS—Fondazione Bietti, Via Livenza 6, 00198 Rome, Italy; vincenzo.parisi@fondazionebietti.it (V.P.); sara.giammaria@fondazionebietti.it (S.G.); lucilla.barbano@fondazionebietti.it (L.B.); lucia.tanga@fondazionebietti.it (L.T.); manuele.michelessi@fondazionebietti.it (M.M.); gloria.roberti@fondazionebietti.it (G.R.); carmela.carnevale@fondazionebietti.it (C.C.); carmen.dellaquila@fondazionebietti.it (C.D.); francesco.oddone@fondazionebietti.it (F.O.); 2 Department of Sense Organs, Faculty of Medicine and Dentistry, Sapienza University of Rome, Viale del Policlinico 155, 00161 Rome, Italy; mattia.dandrea@uniroma1.it; 3 DSCMT, Università di Roma Tor Vergata, Viale Oxford 81, 00133 Rome, Italy; gianlucamanni53@gmail.com

**Keywords:** glaucoma, macula, structure function, OCT, mfPhNR

## Abstract

**Objectives:** This study aimed to study the inner retina functional and morphological impairment of retinal ganglion cells (RGCs) from specific macular rings and sectors to identify whether selective macular regions were more vulnerable than others within the 20 central degrees in patients with open-angle glaucoma (OAG). **Methods:** In total, 21 OAG patients [mean age 50.19 ± 7.86 years, Humphrey Field Analyzer (HFA) 24-2 mean deviation (MD) between −5.02 and −22.38 dB, HFA 10-2 MD between −3.07 and −17.38 dB], providing 21 eyes, were enrolled in this retrospective case–control study. And 20 age-similar healthy subjects, providing 20 eyes, served as controls. The multifocal photopic negative response (mfPhNR) response amplitude density (RAD) from concentric rings and macular sectors and ganglion cell layer thickness (GCL-T) assessed by Spectral Domain–Optical Coherence Tomography (SD-OCT) was measured. Mean RAD and GCL-T values were compared between OAG and control ones by ANOVA. In OAG eyes, the relationship between mfPhNR and SD-OCT data was examined by linear regression analysis, and Pearson’s correlation coefficients were computed. **Results:** In considering all rings and sectors, compared to the controls, the OAG group showed a significant (*p* < 0.01) reduction in mean mfPhNR RAD and in GCL-T values with the greatest reduction in the central area. In OAG eyes, a significant (*p* < 0.01) correlation between all mfPhNR RAD and GCL-T values, with significant (*p* < 0.01) correlation coefficients, were found. **Conclusions**: In OAG eyes, RGC dysfunction was detectable by abnormal mfPhNR responses in localized macular areas, mainly in the central one. Localized macular RGC dysfunction was linearly correlated with the GCL morphological changes.

## 1. Introduction

Open-angle glaucoma (OAG) is a progressive neurodegenerative disease characterized by loss of retinal ganglion cells (RGCs) and the involvement of post-retinal visual pathways [1,2,3]. Approximately 40% of RGCs are located within the central 8° of the macula [4], and for this reason, the functional assessment of central RGCs has become of increasing interest even in early stages of the disease [5,6,7,8,9,10].

RGCs function can be assessed by transient and steady-state pattern electroretinogram (PERG) recordings, and the presence of reduced P50-N95 amplitude may suggest early RGCs dysfunction in ocular hypertension (OHT), glaucoma suspicions, or the early manifestation of glaucoma eyes (see, as a review, Gallo Afflitto et al. [11]).

A novel electrophysiological measure for detecting the function of RGCs and their fibers in OAG is the photopic negative response (PhNR); its amplitude is derived from the negative wave recorded after the positive peak of the b-wave in a cone-driven full-field electroretinogram (ff-ERG) over a wide area of the retina [12] and after the P1-positive peak of the focal [13,14] and multifocal electroretinogram (mfERG) over localized retinal areas [15]. PhNR originates mainly from the neural activity of RGCs and their fibers; it is derived from electrophysiological studies in animal models [16,17] and in patients with diseases involving optic nerves and RGCs [18]. However, a contribution to the origin of PhNR from non-neuronal cells, such as Muller glial cells, has also been hypothesized [15,19].

While the PERG provides functional information about the RGCs located in the whole retina, the new multifocal paradigm, named mfPhNR, has been recently introduced to detect RGC function from localized retinal areas in the macula [15].

In our previous published works [20,21], we assessed mfPhNR amplitude responses from localized retinal areas applying different retinal topographies as concentric annular rings centered to the fovea, retinal sectors, or localized areas corresponding to the Early Treatment of Diabetic Retinopathy Study (ETDRS) map configuration. In these studies [20,21], we considered patients with multiple sclerosis and previous history of optic neuritis (MS-ON), with a selective morphological involvement of the inner retina [detected by reduced ganglion cell layer (GCL) macular thickness, but with normal outer retina macular thickness]. We tested if the obtained mfPhNR responses correlated with the almost corresponding thinning of the GCL macular layers in rings, sectors, or ETDRS areas.

The presence of a significant linear correlation between morpho-functional parameters in the examined areas might suggest that the integrity of the mfPhNR-localized response is influenced by the inner retinal morphology also in humans with neurodegenerative process of the optic nerve [21,22,23].

Morphologically, previous optical coherence tomography (OCT) studies identified that the inferotemporal macular sector is the most susceptible to glaucomatous damage [24,25]. Indeed, macular morphological impairment usually involves one of the two hemiretinas (superior or inferior), in accordance with the topographic distributions of the RGC axons [26].

Considering the absence of previous works on the functional and morphological condition of macular RGCs in glaucoma, assessed by mfPhNR and OCT, in localized and almost corresponding localized areas, we aimed to assess whether in OAG eyes RGC dysfunction was detectable in specific macular areas and whether some of them were functionally more vulnerable than others. In addition, we aimed to verify whether, in the same localized macular areas, the potential RGC dysfunction would be correlated or not with morphological GCL changes.

## 2. Materials and Methods

### 2.1. Participants

All research procedures described in this work adhered to the tenets of the Declaration of Helsinki. The study protocol was approved by the local Ethics Committee (Comitato Etico Territoriale Lazio Area 5, IRCCS Istituti Fisioterapici Ospitalieri, Roma, Italy, Protolocol number 24/FB/24) and upon recruitment, informed consent after the full explanation of the procedure was obtained from each subject enrolled in this study.

A large population of 283 patients affected by OAG and 70 normal age-similar subjects were recruited for this study.

Controls and OAG patients underwent extensive ophthalmologic characterization, including a best-corrected visual acuity (BCVA) measurement evaluated using the modified ETDRS table (Lighthouse, Low Vision Products, Long Island City, NY, USA), slit-lamp biomicroscopy, intraocular pressure (IOP) measurements, indirect ophthalmoscopy, optic nerve head 30° color standard photography, and Humphrey 24-2 and 10-2 automated visual field tests (Humphrey Field Analyzer (HFA) 740; Zeiss, San Leandro, CA, USA).

Normal subjects had an IOP lower than 18 mmHg; BCVA of 0.0 logarithm of the minimum angle of resolution (logMAR) with a refractive error between +2.00 and −2.00 spherical equivalent; and a 24-2 threshold visual field with a mean deviation (MD) of 0.5 decibels (dB) and corrected pattern standard deviation (SD).

Inclusion criteria for OAG patients were the following:

(1) Age between 30 and 75 years;
(2) IOP > 22 mmHg (average of the two highest readings of the daily curve, from 8:00 a.m. to 6:00 p.m., six independent readings, one every two hours) without medical treatment;
(3) Diagnosis of OAG with a repeatable HFA 24-2 SITA Standard and visual field defect compatible with glaucoma, with an MD between −2 and −25 dB;
(4) Typical glaucomatous optic nerve head appearance.

The exclusion criteria for OAG and Controls were the following:

(1) BCVA less than 0.0 logMAR with a refractive error between +2.00 and −2.00 spherical-equivalent diopters.
(2) IOP values greater than 18 mmHg. For OAG patients, the IOP was considered under topical hypotensive treatment (monotherapy as well as combined therapy) during, at least, the eight months preceding the electrophysiological and morphological evaluation. This is because it is known that the RGC function could be modified by a reduction in the IOP with beta-blocker treatment [27,28,29].
(3) Ocular surgery, including cataract surgery, in the last 3 months.
(4) Cataract or macular diseases.
(5) Argon Laser Trabeculoplasty in the last 6 months.
(6) Secondary OHT, including steroid-induced OHT.
(7) Ocular or systemic diseases that could affect the outcome of the study.
(8) Changes in systemic treatments that could affect IOP values.
(9) Pregnancy, breast feeding.
(10) Diabetes.
(11) Systemic lupus erythematosus, rheumatoid arthritis, and connectivitis.

Based on the inclusion/exclusion criteria, 21 consecutive OAG patients (age: mean, 50.19 ± [1 Standard Deviation (1SD)] 7.86 years; range, 36–62 years; HFA 24-2 MD between −5.02 and −22.38 dB; HFA 10-2 MD between −3.07 and −17.38 dB), providing 21 eyes, were selected for the study. And 20 age-similar subjects (age: mean, 51.78 ± (1 SD) 8.82 years; range, 36–62 years; HFA 24-2 and HFA 10-2 with MD between 0.45 and −0.50 dB), providing 20 eyes, served as controls.

### 2.2. Instrumentation and Procedures

#### 2.2.1. Multifocal Photopic Negative Responses Recordings

The mfPhNR was binocularly recorded using a modified version of the Espion system (Diagnosys UK, Ltd.; Histon, Cambridge, UK), as reported in our previous works [20,21].

Pupils were maximally pharmacologically dilated with 1% tropicamide. The pupil diameter (from 7 to 8 mm) was checked by an observer (L.B.) by means of a ruler for each tested eye. The cornea was anesthetized with benoxinate 0.4% eye drops.

The multifocal stimulus consisted of a circular stimulus of 60 elongated scaled dart pattern elements displayed on a monitor (screen size: 69 cm width and 38 cm height), with a mean background luminance of 200 cd/m^2^, at a viewing distance of 33 cm. The stimulus frequency was 7 Hz. Each displayed element was independently reversed between black (0 cd/m^2^) and white (400 cd/m^2^) according to an m-sequence of 12 bits.

The total recording time was on average 20 min of several periods of about 30 s each. Between recording periods, the subject was allowed to rest for a few seconds.

The monitor screen presented a central fixation target, positively perceived by each subject. The eye’s position, fixating on the target, was monitored by a video system on the screen of a computer.

An active Dawson Trick Litzkow bipolar contact electrode was used, and two Ag/AgCl skin electrodes placed on the corresponding outer canthi were used as reference electrodes. Another small Ag/AgCl skin electrode was placed in center of the forehead as the ground. Interelectrode resistance lower than 3 KOhms was considered acceptable.

The signal was filtered (band pass, 3–100 Hz) by the Espion system, and after the automatic rejection of artifacts, the first-order kernel response was considered.

The obtained mfPhNR traces were analyzed, and the average response amplitude densities (RAD) of the mfPhNR expressed in nanoVolt/degree^2^ (nV/deg^2^) were measured from baseline to trough. This is the difference between the pre-stimulus baseline and the more negative point in the trough with an implicit time between 50 and 90 milliseconds (ms) from the stimulus onset.

**Figure 1:**
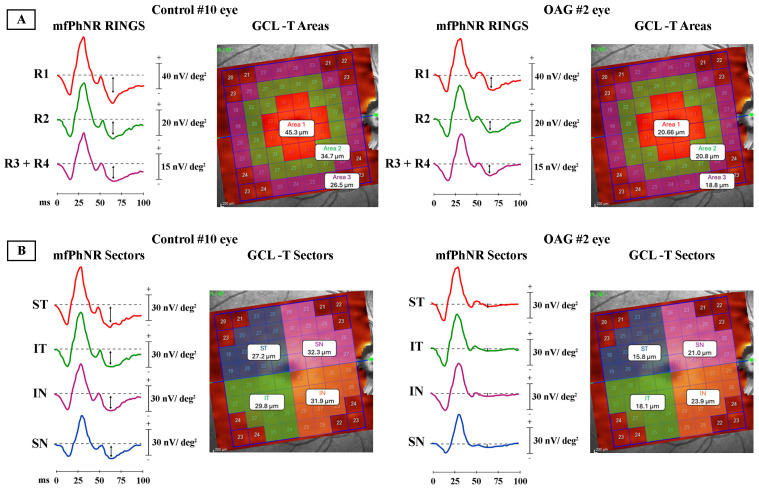
Representative example of functional analysis of retinal ganglion cells (RGCs) assessed with multifocal photopic negative response (mfPhNR) and morphological analysis of ganglion cell layer thickness (GCL-T) in one control eye (#10) and in one open-angle glaucoma eye (OAG#2). (**A**) Multifocal PhNR rings and OCT areas. mfPhNR response amplitude density (RAD) measured in nanoV/degree^2^ (nV/deg^2^) as baseline to trough with an implicit time between 50 and 90 milliseconds (ms) from the stimulus onset and indicated by an arrow (↕). Ring (R) analysis of averaged traces obtained from three circular areas with increasing eccentricity from the fovea, covering 0°–20° from the center. The traces derived from a circular area centered on the fovea with a radius of 5° are depicted in red [Ring 1 (R1)]; in green, an external annular area enclosed between 5° and 10° of foveal eccentricity [Ring 2 (R2)]; in purple, a more external annulus combining together the areas enclosed between 10° and 15° Ring 3 (R3)] and between 15° and 20° [Ring 4 (R4)] (R3 + R4). GCL-T data: the central area corresponds to Area 1 encompassing the superpixels in a 6.35° radius centered on the fovea; the annular parafoveal area corresponds to Area 2, analyzing the superpixels enclosed between 6.35° and 9.37° from the fovea; the annular perifoveal area corresponds to Area 3, analyzing the superpixels enclosed between 9.37° and 12.5° from the fovea. In each area, the GCL-T value is reported in micron (µm). (**B**) Multifocal PhNR and OCT sectors. mfPhNR response amplitude density (RAD) [measured in nanoV/degree^2^ (nV/deg^2^)] obtained in each macular sector. The first sector (S1) corresponds to R1 and analyzes a circular area centered on the fovea with a radius of 5°. The superior temporal (ST), inferior temporal (IT), inferior nasal (IN), and superior nasal (SN) sectors were quarters of an annulus within 5° (inner border) and 20° (outer border) of eccentricity from the fovea, corresponding to the sum of R2 + R3 + R4. The averaged R2 + R3 + R4 RADs for each sector (ST, IT, IN, SN) were measured. GCL-T data report the averaged values from four macular sectors (ST in blue; IT in green; IN in orange; SN in purple), determined by the horizontal and vertical midlines of the posterior pole and intersection at the fovea. The ST, IT, IN, and SN sectors comprise 13 superpixels each, corresponding to the ST, IT, IN, and SN portion of the posterior pole, respectively. GCL-T measurements are provided in microns (µm). OAG#2 eye shows reduced mfPhNR RADs and GCL-T in all rings and sectors when compared to Control#10 eye.

The recorded mfPhNR responses were analyzed using two different topographies centered on the fovea, as follows (see Figure 1):

(1) Ring analysis: we used the same analysis proposed in other reports for mfPhNR responses [20,21].

This was carried out with five concentric annular areas (rings, R) with increasing eccentricity from the fovea: the first one analyzed a circular area centered on the fovea with a radius of 5° (ring 1, R1), the second one analyzed the external annular area enclosed between 5° and 10° of foveal eccentricity (ring 2, R2), the third one analyzed the more external annular area enclosed between 10° and 15° (ring 3, R3), and the fourth one analyzed the annular area between 15° and 20° (ring 4, R4). The averaged RAD values between R3 + R4 was considered in the analysis of results.

(2) Sectors analysis: following previously described analysis for mfPhNR responses [20], we analyzed five sectors covering an area of 20° of eccentricity from the fovea. The first sector (S1) corresponds to R1 (see above), analyzing responses a circular area centered on the fovea with a radius of 5°; the more external sectors (corresponding to R2 + R3 + R4) were quarters of annulus, localized in the superior temporal (ST), superior nasal (SN), inferior nasal (IN), and inferior temporal (IT) areas with respect to the fovea. The annular area (divided into sectors) had a foveal eccentricity from 5° (inner border) to 20° (outer border). The averaged R2 + R3 + R4 RADs for each sector (ST, SN, IN, IT) were measured.

#### 2.2.2. Optical Coherence Tomography

The morphological evaluation of inner retina was carried out by performing Spectral Domain–Optical Coherence Tomography (SD-OCT) automatic segmentation of GCL using a Spectralis OCT device (Heidelberg Engineering, Heidelberg, Germany). SD-OCT scans were obtained in a dark room after pupil dilation with tropicamide 1% eye drops, and each scan was carefully reviewed for the accurate identification and segmentation of the retinal layers by two expert graders (L.B., S.G.) to exclude cases of failed segmentation. Quality control and APOSTEL recommendations according to the published criteria were followed [30].

The OCT image quality signal strength index of the acquired scan was at ≥25. Scans that did not fulfill the above criteria were excluded from the analysis. Images were acquired with the Glaucoma Module Premium Edition software (version 6.16; GMPE, Heidelberg Engineering), consisting of 61 horizontal B-scans (each containing 768 A-scans) averaged 9 times, within the central 30° × 25° of the macula. The automatic segmentation of GCL thickness (GCL-T) measurements was obtained. The GCL-T in the superpixels of the posterior pole analysis grid, approximatively overlapping with the mfPhNR analysis maps, was averaged to obtain the thickness from two topographical regions (see Figure 1):

(1) Ring analysis: the central area (named Area 1) encompassed the superpixels in a 6.35° radius centered on the fovea, Area 2 analyzed the superpixels enclosed between 6.35° and 9.37° from the fovea, and the third area (Area 3) analyzed the superpixels enclosed between 9.31° and 12.5° from the fovea;
(2) Sectors analysis: identified four sectors determined by the horizontal and vertical midlines of the posterior pole and intersecting at the fovea. The ST, IT, SN, and IN sectors comprised 13 superpixels each, corresponding to the ST, IT, SN, and IN portion of the posterior pole, respectively.

In the macular area, the RGCs are radially displaced from the respective photoreceptors with a maximum of approximatively 2° from the fovea in the central 6° of the macula and negligible displacement in the most peripheral macular area [4,31]. The central OCT (Area 1) and mfPhNR (Ring 1), with a radius of 5° and 6.35° from the fovea, respectively, account for most of the displaced RGCs.

### 2.3. Statistical Analysis

We assumed a Gaussian distribution of our data. The normal distribution was assessed using the Kolmogorov–Smirnov test.

Sample size estimates were obtained from pilot evaluations performed in 10 eyes from 10 OAG patients and 10 eyes from 10 control subjects other than those included in the current study. The sizing was based on the following mfPhNR R1 RAD values: 24.7 ± 7.6 nV/deg^2^ for controls and 15.8 ± 6.8 nV/deg^2^ for OAG patients at α = 5% (type 1 error) and power = 80% (β = 20%), giving us 12 participants for each group. Descriptive statistics are shown as the mean and SD. Morphological and electrophysiological data from controls and OAG patients were compared using one-way analysis of variance (ANOVA) and considering groups as factor.

The controls’ data at a 95% lower limit were used to highlight how many OAG eyes showed abnormal GCL-T and mfPhNR RAD values. The 95% confidence limits (CLs) were obtained from the control data.

One eye of each patient was included in the analysis. If both eyes of a patients were eligible for this study, the right eye was included.

Linear regression models were used to describe the changes in the mfPhNR RAD values (dependent variable) across rings from 1 to 3 + 4 and GCL-T values across areas from 1 to 3, respectively (independent variables), with groups as an interaction term.

In OAG patients, linear regression analysis was also used to study the relationship between mfPhNR and SD-OCT data in the corresponding rings/areas and sectors.

Lastly, electrophysiological mfPhNR data were correlated with the corresponding morphological SD-OCT values, and Pearson’s correlation coefficients were computed to assess the strength of these morpho-functional relationships.

A *p* value less than 0.01 was considered statistically significant. SPSS (version 25), MedCalc V.13.0.4.0 (MedCalc, Mariakerke, Belgium) and R (V.4.3.1) [32] software were used for statistical analysis.

## 3. Results

No statistically significant differences were found between the groups’ age [OAG: 21 eyes; age: mean, 50.19 ± 7.86 years. Controls: 20 eyes; age: mean, 51.78 ± 8.82 years (f = 0.37; *p* = 0.545)].

The individual electrophysiological (multifocal photopic negative response amplitude densities) and morphological (ganglion cell layer thickness) values from OAG eyes detected in macular rings/areas or sectors are reported in Table 1.

### 3.1. Multifocal PhNR and OCT Data in Rings and Areas

Figure 1A shows a representative example of a functional analysis of RGCs assessed with the mfPhNR of localized retinal areas (rings) and the corresponding morphological analysis of GCL-T assessed with SD-OCT in one control eye (#10) and in one OAG eye (#2).

The mean data of mfPhNR RAD and GCL-T detected in the control and OAG groups and relative statistical analysis are reported on Table 2(A).

When considering individual values, all OAG eyes showed abnormal values of mfPhNR RADs detected in Ring 1, Ring 2, and Ring 3 + 4 and of GCL-T detected in Area 1 and in Area 2. The GCL-T detected in Area 3 was abnormal on 20/21 (95.2%) OAG eyes (see Table 1 and Table 2(A)).

On average, in the OAG group, a significant (*p* < 0.01) reduction in the mfPhNR RAD mean values detected in Ring 1, Ring 2, and Ring 3 + 4 and a significant (*p* < 0.01) reduction in GCL-T detected in Area 1, Area 2, and Area 3 were observed when compared to the control group. This is reported in Table 2.

**Figure 2:**
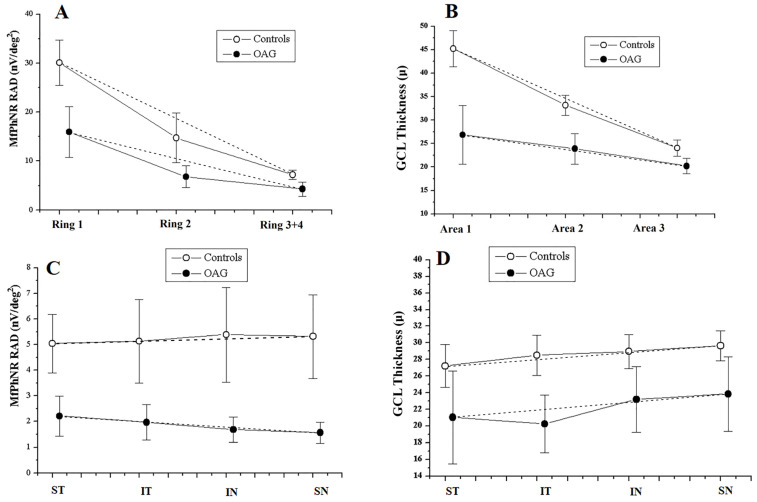
Relationship between multifocal photopic negative response (mfPhNR) response amplitude density (RAD) and ganglion cell layer thickness (GCL-T) values detected in rings and sectors in open-angle glaucoma (OAG) eyes and controls. (**A**) The multifocal photopic negative response (mfPhNR) average response amplitude densities (RADs) measured in controls and OAG eyes recorded in Ring 1, Ring 2, and Ring 3 + Ring 4. (**B**) The ganglion cell layer thickness (GCL-T) values measured in controls and OAG eyes recorded in Area 1, Area 2, and Area 3. (**C**) The multifocal photopic negative responses (mfPhNR) average response amplitude densities (RADs) measured in controls and OAG eyes recorded in the superior temporal (ST), inferior temporal (IT), inferior nasal (IN), and superior nasal (SN) sectors. (**D**) The ganglion cell layer thickness (GCL-T) values measured in controls and OAG eyes recorded in the superior temporal (ST), inferior temporal (IT), inferior nasal (IN), and superior nasal (SN) sectors. In OAG eyes, the averaged mfPhNR RAD and GCL-T values were lower compared to those in the controls but with a similar decreasing pattern at increasing eccentricity (from Ring 1 to Ring 3 + Ring 4 and from Area 1 to Area 3), which was particularly evident in controls (**A**,**B**). Similarly, in OAG eyes, the averaged mfPhNR RAD and GCL-T values were lower compared to in controls in all sectors. However, no significant pattern of changes was found for both averaged mfPhNR RAD and GCL-T values (**C**,**D**).

In Figure 2A,B, the mean values of mfPhNR RAD and GCL-T detected in control and OAG eyes are plotted as a function of retinal eccentricity, respectively. Controls showed significantly higher mfPhNR and GCL-T values in all rings and areas. In control and OAG eyes, a progressive reduction in both mean mfPhNR RAD and GCL-T values occurs from R1 to R3 + R4 and from Area 1 to Area 3, respectively (mfPhNR: OAG: y= −5.20x + 15.95; controls: y= −9.28x + 29.59. GCL-T: OAG: y= −3.32x + 26.85; controls: y= −10.60x + 45.22), with statistically significant decrease in controls (*p* < 0.001 for both models).

**Figure 3:**
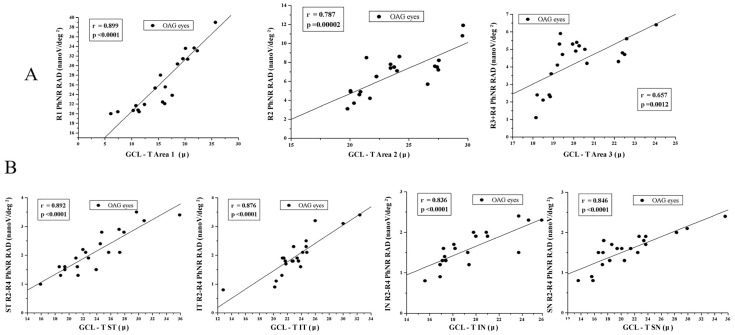
Correlations between mutifocal photopic negative response (mfPhNR) and ganglion cell layer thickness (GCL-T) individual values detected in open-angle glaucoma (OAG) eyes. (**A**) The individual GCL-T values measured in OAG eyes in Area 1, Area 2, and Area 3 were plotted as a function of the corresponding values of the mfPhNR response amplitude densities (RADs) recorded in Ring 1, Ring 2, and Ring 3 + Ring 4. (**B**) The individual GCL-T values measured in OAG eyes in superior temporal (ST), inferior temporal (IT), inferior nasal (IN), and superior nasal (SN) sectors were plotted as a function of the corresponding values of the mfPhNR RADs recorded in the Ring 2 + Ring3 + Ring 4 (R2–R4) ST, IT, IN, and SN sectors. In OAG eyes, the reduction in mfPhNR RADs was significantly correlated to the reduction in GCL-T in all examined rings or sectors.

As shown in Figure 3A, in OAG eyes, the reduction in mfPhNR RADs detected in Ring1, Ring 2, and Ring 3 + Ring 4 was significantly (*p* < 0.01) correlated to the reduction in GCL-T observed in Area 1, Area 2, and Area 3, respectively. MfPhNR RADs in Ring 1, Ring 2, and Ring 3 + Ring 4 and the GCL-T in Area 1, Area 2, and Area 3 were positively related.

The highest values of R^2^ were found between the mfPhNR RAD R1 and GCL-T of Area 1 (R^2^ = 0.80) (Table 3).

### 3.2. Multifocal PhNR and OCT Data in Sectors

Figure 1B shows a representative example of functional analysis of RGCs assessed with the mfPhNR of macular sectors and the corresponding morphological analysis of GCL-T assessed with SD-OCT in one control eye (#10) and in one OAG eye (#2).

The mean data of mfPhNR RAD and GCL-T obtained from macular sectors in the control and OAG groups and the relative statistical analysis are reported in Table 2(B).

When considering individual values, all OAG eyes (22/22, 100%) showed abnormal values of mfPhNR RAD in all sectors, and of GCL-T detected in the IT sector. The GCL-T was abnormal in 15/22 (71.41%) OAG eyes in the SN sector and in 19/22 (90.40%) OAG eyes in the ST and IN sectors (see Table 1 and Table 2(B)).

On average, as reported in Table 2(B), the mean values of mfPhNR RAD and of GCL-T observed in the OAG group were significantly (*p* < 0.01) reduced when compared to the control ones.

In Figure 2C,D, the mean values of mfPhNR RAD and GCL-T detected in control and OAG eyes were plotted as a function of retinal sectors, respectively. Similarly to the analysis for the retinal eccentricity (rings and areas), controls showed significantly higher mfPhNR RADs and GCL-T values in all sectors. However, no statistically significant differences in the pattern of change in the mfPhNR values from the ST to the SN sectors could be found in the two groups (mfPhNR: OAG: y = −0.22x +1.96; controls: y = 0.10x + 5.12, *p* = 0.06). Also, for the GCL-T analysis, higher values were found in controls with respect to OAG eyes, without any pattern of changes from the ST to the SN sectors (GCL-T: OAG: y = 1.13x + 20.42; controls: y = 0.68x + 26.9, *p* = 0.34).

As reported in Figure 3B, in OAG eyes, the reduction in mfPhNR RAD was statistically significantly (*p* < 0.01) correlated with the reduction in GCL-T in all sectors. Sectorial mfPhNR RADs and the GCL-T values were positively related, with the highest values of R^2^ found between the mfPhNR RAD ST and GCL-T ST (R^2^ = 0.53) (see Table 3).

## 4. Discussion

We aimed to study the inner retina functional and morphological impairment of RGCs in glaucomatous eyes from specific macular areas and sectors by performing mfPhNR recordings and OCT acquisitions, with the purpose of identifying whether selective macular areas were more vulnerable than others within the 20 central degrees in OAG.

We used an innovative proven method [15,20,21,22,23] able to investigate the inner retina function to establish whether RGC dysfunction is correlated or not with morphological GCL impairment for corresponding macular rings and sectors.

### 4.1. Functional Data

A functional evaluation of the bioelectrical activity of the neurons of the inner retinal layers of the macular area (macular RGCs) was previously performed using Focal ERG recordings in response to contrast-reversing gratings and the recording of the Visual Evoked Potential (VEP) recovery time after photostress both in patients with OHT and OAG [33,34]. Both electrophysiological methods, however, could not derive responses from selective macular areas. Indeed, both the Focal ERG amplitude reduction and the prolonged recovery time after the photostress of VEP P100 implicit time provided a measure of the reduced functionality of the inner retinal layers of the whole central retina. Subsequent topographical evaluations by mutifocal ERG [35] in glaucomatous patients also assessed reduced foveal RAD within the five central degrees, leading to the conclusion that a functional impairment of the foveal outer and middle retinal layers cannot be excluded in OAG.

For this reason, we aimed to record the mfPhNR, as conducted previously [15,20,21,22,23], to investigate the inner retinal function for selective macular areas, to test whether this method could detect those areas within the vascular arcades, divided into rings and sectors, more prone to glaucomatous damage.

When we analyzed mean RGC functional data from rings (moving concentrically from the fovea up to 20°), we found in OAG eyes a significant (*p* < 0.01) reduction in mfPhNR RAD mean values from all rings compared to in controls (Table 2(A)).

This approach was then extended to examine the macular sectors dividing the macula area in quarters with a foveal eccentricity from 5° to 20°. Also in this case, a significant reduction (*p* < 0.01) in the mean mfPhNR RADs recorded from the SN-ST-IN-IT sectors was found in OAG eyes, compared to the control ones (Table 2(B)).

These results let us make some functional considerations in our cohort of OAG eyes.

First, in glaucomatous eyes, the innovative functional method of mfPhNR allowed us to detect in localized areas in the macula RGC dysfunction by either considering both concentric areas or macular sectors. This suggests that in our OAG eyes, the detected dysfunction of the macular inner retinal neurons (mainly the RGCs) may be observed considering either rings or sectors and is therefore independent from the topographical distribution of RGCs.

However, when considering the pattern of changes in mean mfPhNR RADs from the center to the periphery of the macula (plotting mfPhNR RAD from R1 to R3 + R4) in OAG eyes with respect to control ones, we observed that RAD reduction was maximal in the central macula (R1) and decreased moving from R1 to R3 + R4 (Figure 2A).

Indeed, from anatomical cornerstones studies [4], we assume that almost 50% of total RGCs are located within 4.5 mm (16°) of the foveal center, and that the highest RGCs density is found in an elliptical ring horizontally oriented that extends between 0.4 and 2 mm from the fovea. Within this extension, the RGC density is equal between the superior and inferior retina, and only by 4 mm (an area that we did not explore), the superior retina presents a higher RGC density than the inferior retina. All this suggests that in our OAG eyes, examined over an extension of 20° (therefore within 5.5 mm), on average, at least 50% of RGCs are functionally impaired, with a higher trend in dysfunction involving mainly the macular central 5° (corresponding to R1) compared to more eccentric macular areas (corresponding to R2 and R3 + R4, from 10° to 20°).

The application of similar settings in OAG has been previously performed by Kaneko et al. [15] and Al-Nosairy et al. [22] who recorded the mfPhNR to study the regional dysfunction of RGCs from either five or three retinal areas in patients affected by glaucomatous optic neuropathy, respectively.

In detail, Kaneko et al. [15] found significantly reduced PhNR amplitude (N2 amplitude) in early, intermediate, and advanced OAG eyes only in the central area (0–6.8° of radius), describing a central regional variation in the PhNR with the severity of glaucoma. In this study [15], the mfPhNR amplitude was similar to controls in the eccentric retinal sectors, superior, inferior, temporal, and nasal, from 6.8° to 20° of radius, even in eyes with advanced glaucoma. In the central areas, the mfPhNR amplitude density changes were positively correlated with the reduction in ganglion cell thickness and the visual field sensitivity.

Al-Noisary et al. [22] also studied the mfPhNR/b-wave ratio from five retinal areas (one central 0–5° and four adjacent sectors from 5° to 24° of radius) in suspected and confirmed glaucoma patients using both fast and slow stimulation conditions. They [22] found that this parameter was unaltered in the central area in both groups compared to the peripheral areas where it was found to be decreased.

Therefore, some conclusions proposed by Kaneko et al. [15] and Al-Nosairy et al. [22] seem to be in contrast with our results, and this might be due to different characteristics of the glaucoma patient populations included in the studies or because different macular areas were considered.

We also analyzed mean RGC functional responses derived from macular sectors (ST, IT, IN, SN) within 5° and 20°, and we found in OAG eyes a significant (*p* < 0.01) reduction in mfPhNR RAD mean values in all sectors compared to the controls (Table 2(B)). When we plotted the mfPhNR RAD values as a function of the sectors’ topography (from ST to SN), we found a difference with controls; however, the pattern of changes was not statistically significant (Figure 2C).

All this suggests that in our OAG eyes, we were not able to identify a macular sector more functionally impaired with respect others. This seems to contrast with the findings of Ishizuka et al. [23], who considered the focal PhNR responses from the fovea and from two semicircular areas (inferior and superior) with a 7.5° of radius in three different groups of OAG patients based on the severity of visual field defects. They found reduced focal PhNR amplitude values in all OAG patients in the central and inferior sector. Nevertheless, due to the different methodology used, it is not possible to draw either consensual or contrasting conclusions with our observations.

### 4.2. Morphological Data

Morphological changes through areas in OAG eyes and controls showed similar trends: The measurements in both groups decreased as the eccentricity of the area increased (Figure 2B). In OAGs, the slope of the line describing the change, however, was smaller compared with that in controls, indicating that this change with respect to eccentricity was less pronounced in this group. This result, however, is consistent with the marked reduction in mean GCL-T in all areas in the OAGs. The difference between control and OAG eyes was higher in Area 1 (45.224 vs. 26.829 µm). This result is in agreement with the evidence that the area within the central 8° of the macula has a physiologically higher density of RGCs, arranged in multiple layers [4], and where the glaucoma can cause a wider range of measurable reduction in the GCL-T.

Additionally, the difference between OAG and control eyes decreased by moving to Area 2 and Area 3 (Figure 2B), where physiologically the density of RGCs is lower. Indeed, outside the central 8° of the macula, the RGCs begin to be arranged from multiple layers to a single layer [4], and the glaucoma damage is less evident. For this reason, the morphological damage in OAG, which mainly consists of a considerable reduction in the GCL-T in Area 1 compared to controls, is less pronounced when considering the more peripheral areas (Area 2 and Area 3) (Table 2(A)). As a result, compared with controls, in OAGs, the line describing the change from Area 1 to Area 3 had a smaller slope (−3.32 vs. −10.60).

On the other hand, the sector analysis showed a marked reduction in GCL-T in the IT and ST sectors compared to the respective nasal sectors in OAGs. The thicknesses in the ST and IT sectors, in fact, being lower than the IN and SN sectors, produced a more positive trend moving from the ST sector to the SN compared to the controls (+1.13 vs. +0.68), although this difference was not statistically significant in our cohort (Figure 2D). Nevertheless, these data are in line with available evidence showing that glaucoma damage occurs more frequently at the superior and inferior poles of the optic nerve [24,36,37], causing progressive axonal degeneration and the death of the corresponding RGCs located in the ST and IT hemimaculas [24,25], respectively.

The greater reduction in GCL-T, particularly in the IT sector that we identified, both as the mean value (Table 2(B)) and individually in all OAGs (abnormal values in 100% of patients), is also in agreement with the well-known greater susceptibility of this specific area to glaucoma damage [24,25].

The sector analysis did not identify a significant trend of loss of GCL-T compared to the analysis by area in OAGs compared to controls. However, it did highlight and confirm data in the literature on the involvement of the temporal versus nasal sectors. The two analysis methodologies provide different but complementary information with respect to glaucoma-related morphological changes. Therefore, given the peculiar topography of glaucoma damage, the sector analysis methodology should be considered as complementary to an eccentricity methodology, namely the analysis by area. In fact, the averaging of the thickness of the two hemimaculas within areas could cause a dilution of the differences between upper and lower hemimacula, captured instead by the sector analysis. Conversely, the sector analysis might not reveal the greater central macular involvement.

### 4.3. Morpho-Functional Correlations

Historically, for both glaucoma specialists and scientists, an interesting question was whether in glaucoma there is a correlation between electrophysiological and morphological impairment. For example, Parisi et al. [38,39] observed that in patients with OHT or OAG, reduced PERG amplitude was significantly correlated with the reduction in Retinal Nerve Fiber Layer (RNFL) thickness. More recently, with the use of full-field PhNR, which assesses the neural activity of RGCs located in the whole retina, interesting correlations were observed in glaucomatous patients between the reduction in the PhNR amplitude and the changes in the RNFL thickness [40,41,42]. Also, in animal models, Wilsey and Fortune [43] detected that macular structural and functional losses were correlated.

Therefore, we found it interesting to correlate macular functional and morphological data obtained in our cohort of OAG eyes.

In detail, in considering individual values in annular areas, a strong statistically significant (*p* < 0.01) linear correlation between mfPhNR reduction in Rings 1, 2, and 3 + 4 and GCL-T in Areas 1, 2, and 3 was found in OAG eyes compared to controls (see Figure 3A). This indicates that in considering almost overlapped annular areas, morpho-functional RGC impairment occurs.

However, when analyzing individual data points in rings (see Table 1), all OAG eyes showed reduced mfPhNR RADs in Ring 1, Ring 2, and Ring 3 + 4 and GCL thinning in Area 1 and Area 2, whereas in Area 3, the 95.2% of OAG eyes showed reduced GCL-T. This means that the best correlation between functional and morphological RGC involvement in our OAG cohort was present within the parafoveal area, whereas in the more eccentric macular area (Area 3), functional inner retinal dysfunction is detectable, in some cases, in the absence of morphological abnormality.

Moreover, when we considered individual values from macular sectors, the reduction in mfPhNR RADs was significantly (*p* < 0.01) linearly related to the reduction in GCL-T in OAG eyes in all sectors (see Figure 3B). However, also in this case, individual sectorial mfPhNR RAD values were abnormal in all OAG eyes, as well as the GCL-T values in the IT sector, but it was abnormal in 71.41% of OAG eyes in the SN sector and in 90.40% of OAG eyes in the ST and IN sectors. Therefore, also for macular sectors, RGC functional impairment was consistent with the morphological impairment mainly involving the IT sector, whereas in the SN, ST, and IN sectors, in some OAG eyes, RGC dysfunction should occur in the absence of morphological impairment.

Everything detected in our cohort of OAG patients agrees with our previous studies [20,21] performed using a different model of neurodegeneration, MS-ON. Indeed, similarly to the results observed in the present study, greater RGC dysfunction with reduced GCL-T in the central macular area was found also in the MS-ON model, confirming a regional (central) vulnerability of RGCs by post-inflammatory process.

## 5. Conclusions

The present findings suggest that in OAG eyes, abnormal mfPhNR responses from regional rings reflect RGC dysfunction in localized macular annular areas and mainly in the central one. However, an abnormal mfPhNR response was not indicative of prevalent RGC dysfunction in specific macular sectors. Moreover, abnormal mfPhNR responses were also linearly related with GCL thinning in many OAG eyes, although in some of them, RGC dysfunction also occurred in the absence of GCL thinning.

## Figures and Tables

**Table 1:** Individual electrophysiological (multifocal photopic negative response amplitude densities) and morphological (ganglion cell layer thickness) data observed in eyes with open-angle glaucoma (OAG) detected in correspondent macular rings/areas or sectors.

	Rings/Areas						Sectors							
	R1 ^a^/Area 1		R2 ^a^/Area 2		R3 + R4 ^b^/Area 3		R2 + R3 + R4 ^c^ ST ^d^		R2 + R3 + R4 ^c^ IT ^e^		R2 + R3 + R4 ^c^ IN ^f^		R2 + R3 + R4 ^c^ SN ^g^	
	mfPhNR ^h^ RAD ^i^ (nV/deg^2^) ^l^	GCL-T ^m^ (µ) ^n^	mfPhNR ^h^ RAD ^i^ (nV/deg^2^) ^l^	GCL-T ^m^ (µ) ^n^	mfPhNR ^h^ RAD ^i^ (nV/deg^2^) ^l^	GCL-T ^m^ (µ) ^n^	mfPhNR ^h^ RAD ^i^ (nV/deg^2^) ^l^	GCL-T ^m^ (µ) ^n^	mfPhNR ^h^ RAD ^i^ (nV/deg^2^) ^l^	GCL-T ^m^ (µ) ^n^	mfPhNR ^h^ RAD ^i^ (nV/deg^2^) ^l^	GCL-T ^m^ (µ) ^n^	mfPhNR ^h^ RAD ^i^ (nV/deg^2^) ^l^	GCL-T ^m^ (µ) ^n^
OAG#1	10.8	21.7	8.5	21.4	4.7	19.4	1.9	17.4	2.1	17.3	1.4	24.2	1.8	22.8
OAG#2	10.3	20.7	4.6	20.8	2.4	18.8	1.9	15.8	1.6	18.1	1.7	23.9	1.2	21.0
OAG#3	16.3	25.6	7.5	27.4	4.8	22.4	2.4	23.4	2.3	26.4	1.9	24.7	1.8	24.5
OAG#4	14.4	25.3	8.6	24.2	5.9	19.3	1.5	18.6	1.9	21.0	1.9	23.4	1.7	23.9
*OAG#5*	22.3	33.1	8.2	27.6	4.3	22.2	2.8	22.5	3.4	23.7	2.4	*32.4*	1.5	*28.9*
*OAG#6*	20.1	33.6	7.2	27.5	5.0	20.6	2.9	23.7	2.1	19.8	2	24.8	1.7	*29.2*
OAG#7	6.1	20.0	3.7	20.3	2.3	18.8	1.3	17.3	1.9	18.2	1.6	21.5	1.5	21.3
*OAG#8*	20.6	38.1	10.8	29.6	5.4	20.1	3.2	28.3	1.8	16.9	0.9	22.7	2.0	*30.8*
OAG#9	11.4	20.4	4.9	20.9	4.9	20.1	1.5	16.6	1.7	23.7	1.5	21.8	1.5	19.4
*OAG#10*	21.7	33.6	7.6	27.2	5.6	22.6	2.1	20.1	3.2	25.7	2.3	26.0	1.6	*29.3*
*OAG#11*	19.6	31.4	4.2	21.7	5.3	19.3	2.8	*29.9*	0.8	19.3	1.5	12.8	2.1	18.8
OAG#12	12.4	21.9	6.5	22.2	1.1	18.2	2.1	18.3	1.8	17.1	1.3	22.8	1.3	22.4
OAG#13	25.7	39.0	7.1	24.0	2.1	18.5	1.6	21.4	2.3	24.5	1.8	22.9	1.6	18.6
OAG#14	7.4	20.4	3.1	19.8	2.4	18.2	1.0	19.4	1.1	10.0	1.9	20.4	1.0	15.9
OAG#15	11.2	20.7	5.0	20.1	3.6	18.9	2.2	17.2	1.9	17.2	1.6	21.3	1.2	22.0
OAG#16	17.6	23.8	7.8	23.5	4.2	20.6	1.6	15.6	2.5	26.2	2.1	24.7	0.9	21.2
*OAG#17*	16.2	22.1	7.5	23.7	4.7	22.5	2.8	20.5	3.1	19.4	2.2	*30.0*	1.3	24.7
*OAG#18*	18.6	30.3	11.9	29.6	6.4	*24.1*	3.4	*35.6*	0.9	15.6	0.8	20.2	2.4	*35.9*
OAG#19	15.4	28.0	7.4	23.4	5.2	20.3	2.1	23.7	1.3	17.4	1.3	21.2	1.9	25.7
*OAG#20*	20.5	31.3	5.7	26.6	5.3	19.9	3.5	22.7	1.8	16.9	1.2	23.6	1.9	*29.7*
OAG#21	15.8	22.4	4.9	20.1	4.1	19.2	1.6	13.6	1.8	20.9	2.0	21.7	0.8	19.4
CL ^o^	26.92	43.51	12.71	32.20	6.72	23.27	4.41	26.03	4.41	27.40	4.56	28.05	4.59	28.83

^a^ R1, R2, R3, R4 = concentric annular areas (rings) centered on the fovea; R1, 5° radius circular area; R2, annular area enclosed between 5° and 10° centered on the fovea; R3, annular area enclosed between 10° and 15° centered on the fovea; R4, annular area enclosed between 15° and 20° centered on the fovea; ^b^ R3 + R4, annular area enclosed between 10° and 20° centered on the fovea; ^c^ R2 + R3 + R4, annular area enclosed between 5° and 20° centered on the fovea; ^d^ ST, superior temporal sector; ^e^ IT, inferior temporal sector; ^f^ IN, inferior nasal sector; ^g^ SN, superior nasal sector; ^h^ mfPhNR, multifocal photopic negative response; ^i^ RAD, response amplitude density; ^l^ nV/deg^2^**,** nanoV/degree^2^; ^m^ GCL-T, ganglion cell layer thickness; ^n^ µ, micron; ^o^ CL, confidence limit derived from controls. In *italic*, OAG eyes with abnormal mfPhNR RAD and normal GCL-T. In *italic*, the values not reduced and thus considered “as within normal limits” with respect to the CL.

**Table 2:** Mean values of electrophysiological (multifocal photopic negative response amplitude densities) and morphological (ganglion cell layer thickness) assessment detected in controls (C, 20 eyes) and in eyes with open-angle glaucoma (OAG, 21 eyes) in correspondent macular areas or sectors.

	Controls		OAG		ANOVA ^b^: C vs. OAG: f (1, 40)				
(A) Rings or Areas	Mean	1 SD ^a^	Mean	1 SD ^a^	f=	*p* Value	No ^c^	Ab ^d^	%Ab ^d^
mfPhNR ^e^ R1 ^f^ RAD ^g^ (nV/deg^2^) ^h^	30.047	4.616	15.924	5.188	84.49	<0.001	0	21	100
GCL-T ^i^ Area 1 (µ) ^l^	45.224	3.865	26.829	6.207	128.22	<0.001	0	21	100
mfPhNR ^e^ R2 ^f^ RAD ^g^ (nV/deg^2^) ^h^	14.737	5.073	6.795	2.236	42.79	<0.001	0	21	100
GCL-T ^i^ Area 1 (µ) ^l^	33.150	2.160	23.886	3.270	113.34	<0.001	0	21	100
mfPhNR ^e^ R3 + R4 ^m^ RAD ^g^ (nV/deg^2^) ^h^	7.175	0.962	4.271	1.430	57.61	<0.001	0	21	100
GCL-T ^i^ Area 1 (µ) ^l^	24.030	1.707	0.190	1.663	53.23	<0.001	1	20	95.2
**(B) Sectors**									
mfPhNR ^e^ R2 + R3 + R4 ^n^ ST ^o^ RAD ^g^ (nV/deg^2^) ^h^	5.041	1.431	2.200	0.716	65.59	<0.001	0	21	100
GCL-T ^i^ ST ^o^ Sector (µ)^l^	27.200	2.575	21.029	5.288	22.20	<0.001	2	19	90.4
mfPhNR ^e^ R2 + R3 + R4 ^n^ IT ^p^ RAD ^g^ (nV/deg^2^) ^h^	5.127	1.624	1.967	0.687	66.99	<0.001	0	21	100
GCL-T ^i^ IT ^p^ Sector (µ) ^l^	28.486	2.444	19.776	4.104	67.30	<0.001	0	21	100
mfPhNR ^e^ R2 + R3 + R4 ^m^ IN ^q^ RAD ^g^ (nV/deg^2^) ^h^	5.382	1.859	1.681	0.434	78.82	<0.001	0	21	100
GCL-T ^i^ IN ^q^ Sector (µ) ^l^	28.941	2.021	23.190	3.793	36.17	<0.001	2	19	90.4
mfPhNR ^e^ R2 + R3 + R4 ^m^ SN ^r^ RAD ^g^ (nV/deg^2^) ^h^	5.314	1.637	1.557	0.407	103.99	<0.001	0	21	100
GCL-T ^i^ SN ^r^ Sector (µ) ^l^	29.632	1.812	24.067	4.996	22.03	<0.001	6	15	71.4

^a^ 1 SD, one standard deviation; ^b^ ANOVA, analysis of variance; ^c^ No, number of eyes inside the normal limits; ^d^ Ab, number of eyes outside the normal limits; ^e^ mfPhNR, multifocal photopic negative responses; ^f^ R1, R2, R3, R4 = concentric annular areas (rings) centered on the fovea; R1, 5° radius circular area; R2, annular area enclosed between 5° and 10° centered on the fovea; R3, annular area enclosed between 10° and 15° centered on the fovea; R4, annular area enclosed between 15° and 20° centered on the fovea; ^g^ RAD, response amplitude density; ^h^ nV/deg^2^**,** nanoV/degree^2^; ^i^ GCL-T, ganglion cell layer thickness; ^l^ µ, micron; ^m^ R3 + R4, annular area enclosed between 10° and 20° centered on the fovea; ^n^ R2 + R3 + R4, annular area enclosed between 5° and 20° centered on the fovea; ^o^ ST, superior temporal sector; ^p^ IT, inferior temporal sector; ^q^ IN, inferior nasal sector; ^r^ SN, superior nasal sector; normal limits were obtained from control subjects by calculating the 95% lower confidence limit.

**Table 3:** Relationship between mfPhNR and ganglion cell layer thickness detected in open-angle glaucoma (OAG) eyes.

Parameter 1	Parameter 2	Estimate	95% CL ^a^	R^2 b^	*p* Value
mfPhNR ^c^ R1 ^d^ RAD ^e^ (nV/deg^2^) ^f^	GCL-T ^g^ Area 1 (µ) ^h^	0.75	0.57–0.92	0.80	<0.001
mfPhNR ^c^ R2 ^d^ RAD ^e^ (nV/deg^2^) ^f^	GCL-T ^g^ Area 2 (µ) ^h^	0.53	0.33–0.74	0.62	<0.001
mfPhNR ^c^ R3 + R4 ^i^ RAD ^e^ (nV/deg^2^) ^f^	GCL-T ^g^ Area 3 (µ) ^h^	0.56	0.25–0.88	0.43	0.001
mfPhNR ^c^ R2 + R3 + R4 ^l^ ST ^m^ RAD ^e^ (nV/deg^2^) ^f^	GCL-T ^g^ ST ^m^ Sector (µ) ^h^	0.09	0.05–0.14	0.53	<0.001
mfPhNR ^c^ R2 + R3 + R4 ^l^ IT ^n^ RAD ^e^ (nV/deg^2^) ^f^	GCL-T ^g^ IT ^n^ Sector (µ) ^h^	0.10	0.04–0.16	0.39	0.002
mfPhNR ^c^ R2 + R3 + R4 ^l^ IN ^o^ RAD ^e^ (nV/deg^2^) ^f^	GCL-T ^g^ IN ^o^ Sector (µ) ^h^	0.06	0.01–0.11	0.30	0.009
mfPhNR ^c^ R2 + R3 + R4 ^l^ SN ^p^ RAD ^e^ (nV/deg^2^) ^f^	GCL-T ^g^ SN ^p^ Sector (µ) ^h^	0.05	0.02–0.08	0.41	0.001

^a^ 95% CL, 95% lower confidence limit; ^b^ R^2^, determination coefficient; ^c^ mfPhNR, multifocal photopic negative response; ^d^ R1, R2, R3, R4 = concentric annular areas (rings) centered on the fovea; R1, 5° radius circular area; R2, annular area enclosed between 5° and 10° centered on the fovea; R3, annular area enclosed between 10° and 15° centered on the fovea; R4, annular area enclosed between 15° and 20° centered on the fovea; ^e^ RAD, response amplitude density; ^f^ nV/deg^2^**,** nanoV/degree^2^; ^g^ GCL-T, ganglion cell layer thickness; ^h^ µ, micron; ^i^ R3 + R4, annular area enclosed between 10° and 20° centered on the fovea; ^l^ R2 + R3 + R4, annular area enclosed between 5° and 20° centered on the fovea; ^m^ ST, superior temporal sector; ^n^ IT, inferior temporal sector; ^o^ IN, inferior nasal sector; ^p^ SN, superior nasal sector.

## Data Availability

Data supporting the reported results are available upon request to the corresponding author.

## Abbreviations and Acronyms

ANOVA	one-way analysis of variance
BCVA	best-corrected visual acuity
CLs	confidence limits
ETDRS	Early Treatment Diabetic Retinopathy Study
ff-ERG	full-field electroretinogram
GCL	ganglion cell layer
GCL-T	ganglion cell layer thickness
HFA	Humphrey Field Analyzer
IN	inferior nasal
INL	inner nuclear layer
IOP	intraocular pressure
IPL	inner plexiform layer
IT	inferior temporal
logMAR	logarithm of the minimum angle of resolution
MD	mean deviation
mfERG	multifocal electroretinogram
mfPhNR	multifocal photopic negative response
MS-ON	multiple sclerosis-optic neuritis
N	number of eyes of each group
OAG	open-angle glaucoma
OCT	optical coherence tomography
OHT	ocular hypertension
PERG	pattern electroretinogram
PhNR	photopic negative response
R	ring
RAD	response amplitude density
RGCs	retinal ganglion cells
RNFL	retinal nerve fiber layer
SD	one standard deviation of the mean
SD-OCT	spectral domain–optical coherence tomography
SN	superior nasal
ST	superior temporal
VEP	visual evoked potential
